# GAIN2 trial overall survival with intense versus tailored dose dense chemotherapy in early breast cancer

**DOI:** 10.1038/s41523-024-00675-x

**Published:** 2024-07-30

**Authors:** Volker Möbus, Hans-Joachim Lück, Ekkehart Ladda, Peter Klare, Knut Engels, Marcus Schmidt, Andreas Schneeweiss, Eva-Maria Grischke, Grischa Wachsmann, Helmut Forstbauer, Michael Untch, Frederik Marmé, Jens-Uwe Blohmer, Christian Jackisch, Jens Huober, Elmar Stickeler, Mattea Reinisch, Theresa Link, Bruno Sinn, Wolfgang Janni, Carsten Denkert, Sabine Seiler, Christine Solbach, Sabine Schmatloch, Julia Rey, Sibylle Loibl

**Affiliations:** 1 Medizinische Klinik II, Abt. Hämatologie/Onkologie, Universitätsklinikum Frankfurt, Frankfurt, Germany; 2 Gynäkologisch-onkologische Praxis Hannover, Hannover, Germany; 3 Onkologische Praxis Neumarkt, Neumarkt, Germany; 4 Praxisklinik Krebsheilkunde für Frauen, Berlin, Germany; 5 Zentrum für Pathologie, Zytologie und Molekularpathologie Neuss, Neuss, Germany; 6 Universitätsmedizin Mainz, Mainz, Germany; 7 Nationales Centrum für Tumorerkrankungen, Universitätsklinikum und Deutsches Krebsforschungszentrum, Heidelberg, Germany; 8 Frauenklinik, Universitätsklinikum Tübingen, Tübingen, Germany; 9 Frauenklinik, Kreiskliniken Böblingen gGmbH, Böblingen, Germany; 10 GOSPL - Gesellschaft für onkologische Studien Troisdorf, Troisdorf, Germany; 11 Helios Kliniken Berlin-Buch, Berlin, Germany; 12 Universitätsfrauenklinik Mannheim, Mannheim, Germany; 13 Gynäkologie mit Brustzentrum, Charité-Universitätsmedizin Berlin, Berlin, Germany; 14 Sana Klinikum Offenbach, Offenbach, Germany; 15 Universitätsklinikum Ulm, Ulm, Germany; 16 Department of Obstetrics and Gynecology, Center for Integrated Oncology (CIO Aachen, Bonn, Cologne, Düsseldorf), University Hospital of RWTH Aachen, Aachen, Germany; 17 Kliniken Essen-Mitte, Essen, Germany; 18 Klinik und Poliklinik für Frauenheilkunde und Geburtshilfe, Universitätsklinikum Carl Gustav Carus Dresden, Dresden, Germany; 19 Institut für Pathologie, Charité-Universitätsmedizin Berlin, Berlin, Germany; 20 Institute of Pathology, Philipps University Marburg, Marburg University Hospital (UKGM), and University Cancer Center Frankfurt-Marburg (UCT), Marburg, Germany; 21 German Breast Group, Neu-Isenburg, Germany; 22 Brustzentrum, Universitätsklinikum Frankfurt, Frankfurt, Germany; 23 Elisabeth Krankenhaus Kassel, Kassel, Germany

**Keywords:** Breast cancer, Breast cancer

## Abstract

GAIN-2 trial evaluated the optimal intense dose-dense (idd) strategy for high-risk early breast cancer. This study reports the secondary endpoints pathological complete response (pCR) and overall survival (OS). Patients (*n* = 2887) were randomized 1:1 between idd epirubicin, nab-paclitaxel, and cyclophosphamide (iddEnPC) versus leukocyte nadir-based tailored regimen of dose-dense EC and docetaxel (dtEC-dtD) as adjuvant therapy, with neoadjuvant therapy allowed after amendment. At median follow-up of 6.5 years (overall cohort) and 5.7 years (neoadjuvant cohort, *N* = 593), both regimens showed comparable 5-year OS rates (iddEnPC 90.8%, dtEC-dtD 90.0%, *p* = 0.320). In the neoadjuvant setting, iddEnPC yielded a higher pCR rate than dtEC-dtD (51.2% vs. 42.6%, *p* = 0.045). Patients achieving pCR had significantly improved 5-year iDFS (88.7% vs. 70.1%, HR 0.33, *p* < 0.001) and OS rates (93.9% vs. 83.1%, HR 0.32, *p* < 0.001), but OS outcomes were comparable regardless of pCR status. Thus, iddEnPC demonstrates superior pCR rates compared to dtEC-dtD, yet with comparable survival outcomes.

## Introduction

Dose-dense chemotherapy has shown improved outcomes compared to conventionally dosed chemotherapy in breast cancer. Most guidelines, like those of the European Society for Medical Oncology (ESMO), recommend such chemotherapy regimens as standard of care^1^. The recent meta-analysis by the EBCTCG showed that dose-dense administration of chemotherapy consistently improved invasive disease-free survival (iDFS) and overall survival (OS) over conventionally scheduled regimens^2^. However, a direct comparison between the available dose-dense regimens has rarely been conducted, and indirect comparisons (based on the data of the EBCTCG meta-analysis, for example) have been performed. In general, there are fewer data for neoadjuvant dose-dense chemotherapy than for adjuvant treatment.

In recent decades, different approaches have been described to achieve higher dose intensities. Dose-dense (dd), intense dose-dense (idd), and tailored dose-dense (tdd) regimens must be carefully differentiated^3^. All three schedules share granulocyte-colony-stimulating factor (G-CSF) support and shorter treatment intervals (q2w). Dose-dense regimens (q2w) apply the same total dose in comparison with conventionally dosed chemotherapy (q3w)^4,5^, whereas idd regimens also apply a higher total dose per cycle, corresponding to the maximum tolerated dose^6,7^. In contrast, the tdd approach uses adverse events, especially hematologic toxicity, as a pharmacokinetic surrogate for tailoring chemotherapy dose individually^8^. A positive correlation between efficacy and hematological toxicity has been indicated by several trials^9^.

Based on the 10-year OS data of the idd epirubicin (E), paclitaxel (P), cyclophosphamide (C) regimen (iddEPC), which showed an absolute improvement of 10% in patients with ≥4 positive lymph nodes^7^, we consider iddEPC as one standard regimen in patients with high-risk of recurrence. In the GAIN-2 trial, we substituted paclitaxel with nab-paclitaxel (nP), which provides a potentially higher efficacy compared to solvent-based taxanes and might therefore be the preferred taxane in an idd regimen^10,11^. In the neoadjuvant GeparSepto trial, a significantly higher pathological complete response (pCR) rate with nab-paclitaxel translated into a significantly improved iDFS compared with paclitaxel, especially in luminal-like and TNBC subtype^12^. The tdd regimen had shown a non-statistically significant improvement over standard chemotherapy in the PANTHER trial^13^.

Multiple meta-analyses have demonstrated that patients who achieved a pCR regardless of the breast cancer subtype have better survival outcomes compared to those who did not^14–17^.

The GAIN-2 trial assessed whether tailored dose-dense (dtEC-dtD) versus intense dose-dense (iddEnPC) chemotherapy differs with respect to the pCR rate and long-term outcome depending on subtype and nodal status.

## Results

### Baseline characteristics

Between 10/2012 and 07/2017, 3411 patients were screened for eligibility, 2887 were randomized, and 2857 patients started treatment (iddEnPC 1429; dtEC-dtD 1428). Treatment was completed by 1259/1429 (88.1%) patients in the iddEnPC arm and 1258/1428 (88.1%) in the dtEC-dtD arm (*p* = 1.000) (Supplementary Fig. 1).

**Fig. 1:**
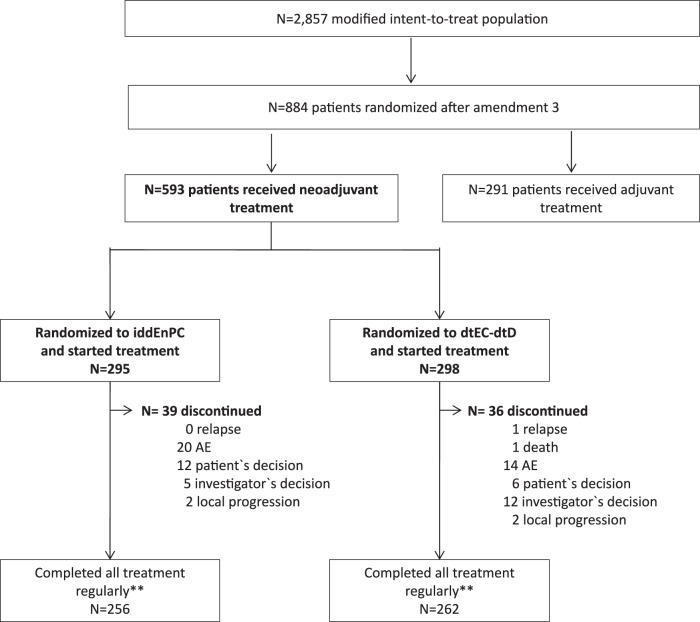
Consort diagram. AE adverse event, dtEC-dtD dose-dense, dose-tailored epirubicin/cyclophosphamide–dose-dense, dose-tailored docetaxel, iddEnPC intense dose-dense epirubicin, nab-paclitaxel, cyclophosphamide; **“Completed treatment” includes patients who completed cyclophosphamide part of the study if treated in the iddEnPC arm and the docetaxel part of the study if treated in the dtEC-dtD arm, respectively, even if previous infusions were skipped.

A total of 884 patients were recruited and treated after amendment 3: 593 patients received neoadjuvant and 291 received adjuvant treatment (Fig. 1). Moreover, 584 (98.4%) patients underwent surgery. A total of 195/291 (67%) neoadjuvant patients in the iddEnPC arm and 201/293 (68.8%) in the dtEC-dtD arm with a documented breast surgery underwent breast conservation surgery (*p* = 0.701).

Baseline patient and tumor characteristics were balanced between the two idd regimens and have been reported previously^18^. Patients treated in the neoadjuvant and adjuvant settings (enrolled after amendment 3) showed pronounced differences in the distribution of risk factors (Table 1). Patients receiving neoadjuvant treatment were younger (57% vs 49.5% premenopausal) and had more aggressive subtypes (HER2+ or TNBC: 74.9% vs 25.4%), less luminal A-like biology (1.9% vs 37.5%), and more high-grade tumors overall (62.7% vs 40.9%).

**Table 1:** Baseline characteristics neoadjuvant vs. adjuvant cohort (patients included after third amendment)

Parameter		Neoadjuvant *N* = 593 *N* (%)	Adjuvant *N* = 291 *N* (%)	Overall *N* = 884 N (%)	*p*-value
Age, years	<30	24 (4.0)	1 (0.3)	25 (2.8)	<0.001
	30–<40	86 (14.5)	28 (9.6)	114 (12.9)	
	40–<50	195 (32.9)	77 (26.5)	272 (30.8)	
	50–<60	208 (35.1)	124 (42.6)	332 (37.6)	
	60–65	60 (10.1)	48 (16.5)	108 (12.2)	
	>65, biologically younger	20 (3.4)	13 (4.5)	33 (3.7)	
Menopausal status	Premenopausal	338 (57.0)	144 (49.5)	482 (54.5)	0.037
	Postmenopausal	255 (43.0)	147 (50.5)	402 (45.5)	
Karnofsky index	80%	3 (0.5)	5 (1.7)	8 (0.9)	0.046
	85%	0 (0.0)	0 (0.0)	0 (0.0)	
	90%	54 (9.1)	37 (12.7)	91 (10.3)	
	95%	0 (0.0)	0 (0.0)	0 (0.0)	
	100%	536 (90.4)	249 (85.6)	785 (88.8)	
Bilateral tumor	No	579 (97.6)	281 (96.6)	860 (97.3)	0.381
	Yes	14 (2.4)	10 (3.4)	24 (2.7)	
Tumor focality	Unifocal	474 (79.9)	183 (62.9)	657 (74.3)	<0.001
	Multifocal	77 (13.0)	66 (22.7)	143 (16.2)	
	Multicentric	42 (7.1)	42 (14.4)	84 (9.5)	
Tumor stage (all)^a^	c/pT1	224 (37.8)	103 (35.4)	327 (37.0)	<0.001
	c/pT2	312 (52.7)	129 (44.3)	441 (49.9)	
	c/pT3	25 (4.2)	53 (18.2)	78 (8.8)	
	c/pT4	31 (5.2)	6 (2.1)	37 (4.2)	
Nodal status (all)^a^	c/pN0-1	514 (86.8)	81 (27.8)	595 (67.4)	<0.001
	c/pN2	60 (10.1)	120 (41.2)	180 (20.4)	
	c/pN3	18 (3.0)	90 (30.9)	108 (12.2)	
ER/PgR	Both ER and PgR negative	253 (42.7)	43 (14.8)	296 (33.5)	<0.001
	ER and/or PgR positive	340 (57.3)	248 (85.2)	588 (66.5)	
sTILs^b^	Low (0–10%)	314 (53.1)	217 (74.6)	531 (60.2)	<0.001
	Intermediate (11–59%)	225 (38.1)	60 (20.6)	285 (32.3)	
	High (60–100%)	52 (8.8)	14 (4.8)	66 (7.5)	
Biological subtype	Luminal A high risk	11 (1.9)	109 (37.5)	120 (13.6)	<0.001
	Luminal B/HER2-	138 (23.3)	108 (37.1)	246 (27.8)	
	Triple negative	172 (29.0)	33 (11.3)	205 (23.2)	
	HER2+ER+ and/or PgR+	191 (32.2)	31 (10.7)	222 (25.1)	
	HER2+ non-luminal	81 (13.7)	10 (3.4)	91 (10.3)	
HER2, central	negative	321 (54.1)	250 (85.9)	571 (64.6)	<0.001
	positive	272 (45.9)	41 (14.1)	313 (35.4)	
Tumor grading	G1	6 (1.0)	5 (1.7)	11 (1.2)	<0.001
	G2	215 (36.3)	167 (57.4)	382 (43.2)	
	G3	372 (62.7)	119 (40.9)	491 (55.5)	
Histological tumor type	lobular invasive	22 (3.7)	49 (16.8)	71 (8.0)	<0.001
	other	571 (96.3)	242 (83.2)	813 (92.0)	
Ki67, central	≤20%	72 (12.1)	123 (42.3)	195 (22.1)	<0.001
	>20%	521 (87.9)	168 (57.7)	689 (77.9)	

^a^Information missing from one patient. For patients receiving neoadjuvant treatment, cT and cN were used. For patients receiving adjuvant treatment, pT and pN were used.

^b^Information missing from two patients.

Baseline patient and tumor characteristics for all patients according to setting (adjuvant or neoadjuvant) are presented in Supplementary Table 1.

### Overall survival (entire cohort)

**Fig. 2:**
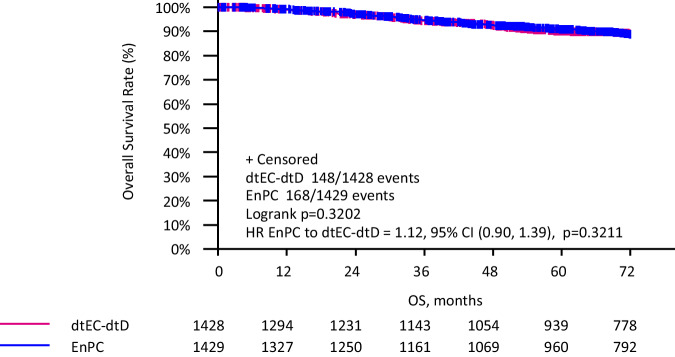
Kaplan–Meier curves for overall survival in both treatment regimens (entire cohort). Survival rates are shown over a 6-year period. The pink curve represents the dtEC-dtD cohort, and the blue curve represents the iddEnPC cohort. CI confidence interval, dtEC-dtD dose-dense, dose-tailored epirubicin/cyclophosphamide–dose-dense, dose-tailored docetaxel, iddEnPC intense dose-dense epirubicin, nab-paclitaxel, cyclophosphamide, HR hazard ratio.

After a median follow up of 6.5 years, 29 patients died in the iddEnPC arm vs 24 in the dtEC-dtD arm. OS was comparable in the two treatment arms: the 5-year OS was 90.8% (95% CI 89.1–92.3.) in the iddEnPC arm and 90.0% (95% CI 88.2–91.6) in the dtEC-dtD arm (HR = 1.12, 95%CI 0.90–1.39; *p* = 0.320) (Fig. 2). In line with the OS, all other secondary long-term endpoints (distant disease-free survival, locoregional relapse-free interval, and local relapse-free interval) showed no differences between treatment arms (data not shown). Analysis of OS by subgroups (biological subtype, Ki67, nodal status, treatment setting, sTILs, and pCR) showed no significant differences between both regimens in any of the predefined subgroups (Supplementary Fig. 2).

### Efficacy – pCR (neoadjuvant cohort)

Short-term efficacy endpoints of pCR (ypT0/is ypN0) and breast conservation rate have been analyzed for patients who received neoadjuvant treatment (*N* = 593).

pCR was achieved by 151 patients (51.2%) in the iddEnPC arm and 127 patients (42.6%) in the dtEC-dtD arm (*p* = 0.045) with an absolute difference of 8.6% (95% CI 0.6–16.6%). Multivariable logistic regression analysis adjusted for stratification factors confirmed that treatment was an independent predictor for achievement of pCR, with OR iddEnPC vs dtEC-dtD: 1.48 (95% CI 1.03–2.12, *p* = 0.033) (Supplementary Table 2).

**Fig. 3:**
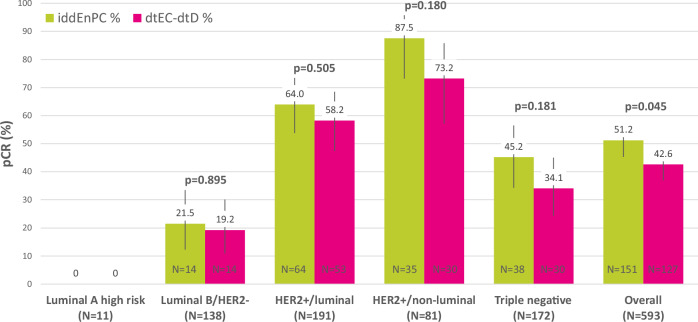
pCR rate in the breast (ypT0/is ypN0) depending on treatment and subtype. pCR rates are presented as separate percentages for the five different subtypes, as well as overall for all subtypes stratified according to treatment arm (iddEnPC in green and dtEC-dtD in pink). Numbers of patients for each subtype (according to treatment arm) are included at the bottom of each bar. Total numbers of patients per tumor subtype and overall are included in the *x*-axis. *P* values are reported for each subtype to evaluate any statistically significant differences between pCR rates in both treatment arms (*p* value <0.05 is considered statistically significant).

pCR rates by treatment arm and subtype are shown in Fig. 3. Luminal A subtype included only 11 patients, none of whom achieved a pCR, and this subtype is not further discussed. All other subtypes were associated with a higher pCR rate with iddEnPC compared to the dtEC-dtD regimen. A total of 28/138 patients with luminal B/HER2- tumors achieved a pCR (20.3%), 117/191 with HER2+, ER and/or PR+ (61.3%), 65/81 with HER2+/HR− (80.2%), and 68/172 with TNBC subtypes (39.5%) achieved a pCR.

Results for pCR in stratified and prospectively defined subgroups are presented in Supplementary Fig. 3. When comparing pCR between both arms within individual subgroups, the group of cN0-1 patients (OR iddEnPC vs dtEC-dtD: 1.50; 95% CI 1.06–2.13, *p* = 0.022) and the group with a Ki67 of more than 20% (OR iddEnPC vs dtEC-dtD: 1.46; 95% CI 1.03–2.06, *p* = 0.033) had significantly higher pCR rate with iddEnPC compared to dtED-dtD. However, tests for interaction between treatment and subgroup parameters were not significant, no subgroup revealed a significantly different treatment benefit.

### Efficacy – pCR and survival (neoadjuvant cohort)

After a median follow up of 5.7 years, 75 patients in the neoadjuvant cohort had an iDFS event (*n* = 18 invasive locoregional relapses, *n* = 2 contralateral relapses, *n* = 70 distant relapses, *n* = 17 secondary malignancies, and *n* = 11 deaths).

**Fig. 4:**
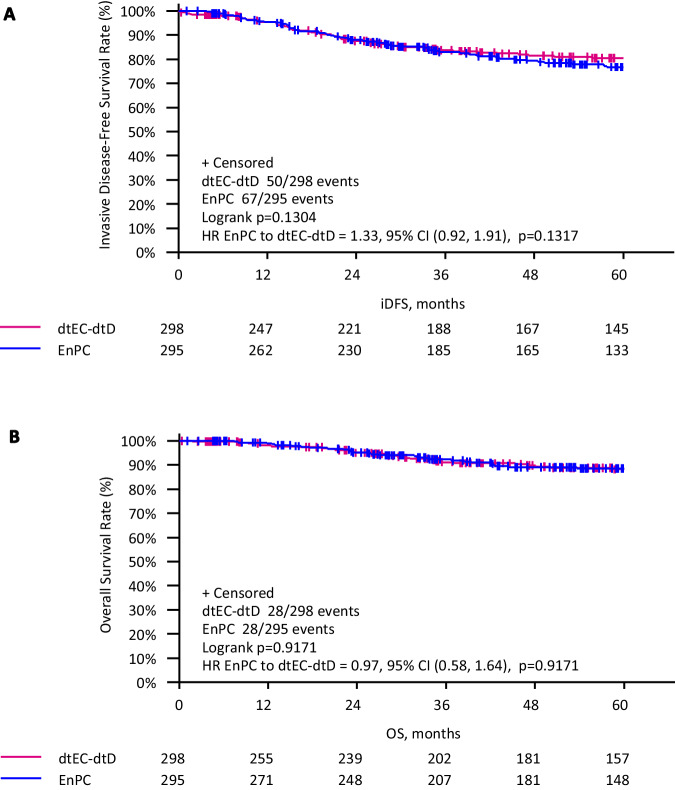
Kaplan–Meier curves. Kaplan–Meier curves **A** iDFS and **B** OS between treatment arms in the subgroup of neoadjuvant patients (mITT-set). Survival rates are shown over a 5-year period. The pink curve represents the dtEC-dtD cohort, and the blue curve represents the iddEnPC cohort.

The 5-year iDFS in the neoadjuvant cohort was 76.7% (95% CI 70.8–81.5%) in the iddEnPC arm and 80.4% (95% CI 74.8–84.8%) in the dtEC-dtD arm, and there was no statistically significant difference in iDFS rates between both treatment arms (Fig. 4a) (HR 1.33; 95% CI 0.92–1.91, *p* = 0.132). The 5-year OS in the neoadjuvant cohort was 88.5% (95% CI 83.8–92.0%) in the iddEnPC arm and 88.2% (95% CI 83.3%–91.7%) in the dtEC-dtD arm, and, similarly, there was no statistically significant difference in OS rates between treatment arms (Fig. 4b) (HR 0.97; 95% CI 0.58–1.64, *p* = 0.917).

**Fig. 5:**
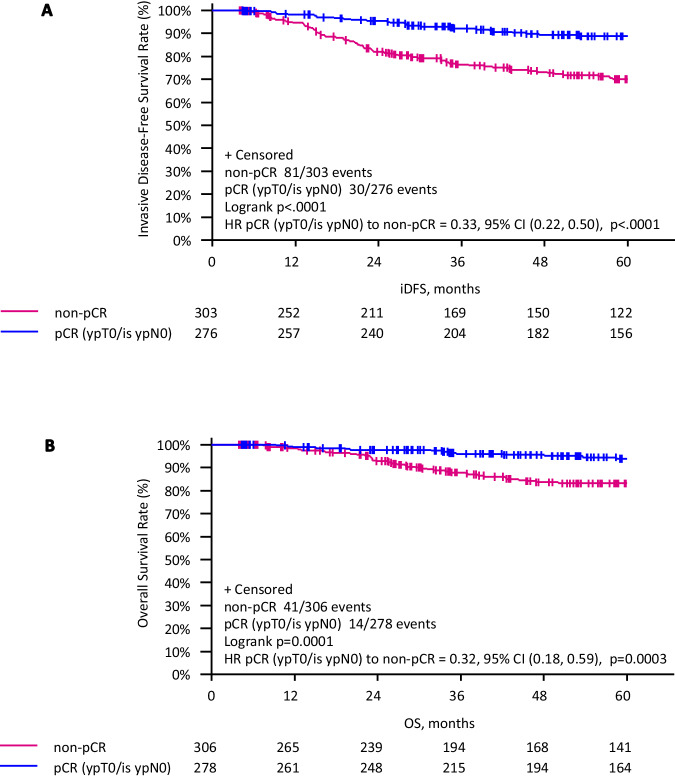
Kaplan–Meier curves. Kaplan–Meier curves for **A** iDFS and **B** OS between patients with pCR vs. without pCR arms in the subgroup of neoadjuvant patients (mITT-set). Survival rates are shown over a 5-year period. The pink curve represents patients with non-pCR, and the blue curve represents patients with pCR (ypT0/is ypN0).

Achieving pCR vs. non-pCR resulted in a significantly improved iDFS (Fig. 5a). 5-year iDFS was 88.7% in patients with pCR versus 70.1% in patients with no pCR (HR 0.33; 95% CI 0.22–0.50, *p* < 0.001). Both regimens showed similar efficacy independent from pCR status, as the 5-year iDFS in patients with pCR was 86.7% with iddEnPC and 91.0% with dtEC-dtD (HR 1.75; 95% CI 0.82–3.74, *p* = 0.144), and in patients without pCR, 5-year iDFS was 66.2% with iddEnPC and 73.7% with dtEC-dtD (HR 1.50; 95% CI 0.97–2.33, *p* = 0.068).

OS was significantly improved in patients achieving a pCR compared to those who did not (Fig. 5b). 5-year OS was 93.9% in patients with pCR versus 83.1% in patients with no pCR (HR 0.32; 95% CI 0.18–0.59, *p* < 0.001). Both regimens showed similar efficacy according to pCR status, as the 5-year OS in patients with pCR was 94.3% with iddEnPC and 93.5% with dtEC-dtD (HR 0.88; 95% CI 0.31–2.50, *p* = 0.806), and in patients without pCR, 5-year OS was 82.1% with iddEnPC and 84.1% with dtEC-dtD (HR 1.15; 95% CI 0.62-2.13, *p* = 0.652).

### Exploratory analysis according to setting after Amendment 3

Results comparing adjuvant versus neoadjuvant treatment for the cohort of patients recruited after amendment 3 require careful interpretation, as patients were not randomly assigned, and selection bias has likely contributed to the aforementioned differences in risk profiles (Table 1).

Exploratory analyses of iDFS and OS in the neoadjuvant and adjuvant settings were performed according to biological subtype (Supplementary Figs. 4, 5). Multivariate regression analysis of OS and iDFS in patients recruited after amendment 3 revealed that TNBC subtype and c/pN2 and c/pN3 nodal status were independent predictors of shorter OS and iDFS, and the HER2+, ER and/or PR+ subtype was an independent predictor of a longer OS (Supplementary Figs. 6, 7).

## Discussion

The GAIN-2 trial compared two different approaches to deliver dose-dense chemotherapy either as adjuvant or neoadjuvant treatment in patients with early node-positive or high-risk node-negative breast cancer. The study was amended to allow the recruitment of patients for neoadjuvant treatment because of slow recruitment. After the third amendment, a total of 593 of 2857 patients were included to receive neoadjuvant chemotherapy. Patients were not randomized between adjuvant or neoadjuvant setting; the allocation was decided by the investigator.

In this analysis, we report that 5-year OS data was similar in both, the intensified dose-dense regimen (iddEnPC) and the toxicity tailored dose-escalation regimen (dtEC-dtD). This is in line with our previous report that showed identical 4-year iDFS rates in both arms, 84.3% (HR 1.01; 95% CI 0.83–1.23, *p* = 0.910)^18^.

Regarding the neoadjuvant cohort, almost half of the patients achieved a pCR (278/593, 46.8%). pCR rates were significantly higher in the iddEnPC arm compared to the dtEC-dTD arm (51.2% vs. 42.6%; *p* = 0.045), with an absolute pCR difference of 8.6% between the treatment regimens. A pCR rate of 20.3% in patients with luminal B/HER2- tumors is remarkable when compared with the recently reported pCR rates in the standard arms of KEYNOTE-756 (15.6%)^19^ and CheckMate 7FL (13.8%)^20^, which included only grade 3 luminal/HER2- tumors. However, the elevated pCR rate with idd treatment did not translate into a superior iDFS or OS in this cohort, most likely due to the rather small sample size of only 593 patients (data not shown). Patients with TNBC receiving idd had a pCR rate of 45%, which is higher than all other non carboplatinum-containing regimens. Unfortunately, data are lacking for PD-1 or PD-L1 inhibitors given with an intensified dose-dense regimen.

The results of the AGO iddEPC trial^7^ showed that iddEPC compared to standard EC/3-weekly paclitaxel was associated with a significantly higher 10-year EFS (56% vs. 47%; HR 0.74, 95% CI 0.63–0.87; *p* = 0.00014) and 10-year OS rates (69% vs. 59%; HR 0.72, 95% CI 0.60–0.87; *p* = 0.0007). As a result, the German national guidelines recommend this regimen as standard of care for patients with high-risk disease and ≥4 axillary lymph node metastases^21^. However, one must acknowledge that the advantage for iddEPC over 3 weekly sequential EC/paclitaxel lacks a control reference to the current standard. While EC with weekly paclitaxel represents the clinically preferred regimen and serves as standard backbone in various chemotherapy trials for breast cancer, a clean comparison with iddEPC has not been reported yet. The trial by Sparano et al.^22^ investigating the efficacy of docetaxel and paclitaxel applied in weekly schedules following 3-weekly doxorubicin/cyclophosphamide demonstrated an advantage for weekly over 3-weekly paclitaxel for DFS (HR 0.84; *p* = 0.011) and a marginal improvement in OS (HR 0.87; *p* = 0.09). Furthermore, a recent meta-analysis by EBCTCG^2^ confirmed a reduced 10-year risk of recurrence (absolute 4.3%) for dose dense regimen and an absolute 2.8% reduction in 10-year breast cancer mortality. It is justified to consider that part of the remarkable absolute increase in OS of 10% observed in the iddEPC AGO trial might be leveled out by the dose-density effects of 2 weekly EC and weekly paclitaxel. The extent of this, however, cannot be quantified, as most trials either investigated the effect of dose-dense EC or weekly paclitaxel or added further chemotherapeutic agents^23,24^. Accordingly, the German national guidelines today specify several different dose-dense regimens for use as adjuvant chemotherapy, with the iddEPC regimen being recommended specifically for high-risk early breast cancer with ≥4 affected lymph nodes.

GAIN-2 and GeparOcto are the only trials which compared two different dose-dense regimens as neoadjuvant therapy, and which combined idd chemotherapy with dual anti-HER2 antibody blockade in the HER2+ subtype. Results of iDFS and OS in non-pCR patients were not influenced by post-neoadjuvant therapies (e.g., T-DM1 in HER2+ or capecitabine in TNBC) in the HER2+ and TNBC subgroups. Most remarkable are the results of the HER2+ subtype in the adjuvant setting. Considering the approval status, all patients received trastuzumab only, and no patients received dual blockade. In GAIN-2, 78.7% of patients treated in the adjuvant setting were node-positive and showed an impressive 4-year OS rate of 92.8% with trastuzumab alone. These results are in line with the results on dual blockade in the adjuvant Aphinity trial^25^, which reported 6-year OS rates of 95% with and 94% without adjuvant pertuzumab. Although the percentage of axillary node-positive disease was identical between both trials, patients with pN2/pN3 node positive disease were more than twice as frequent in GAIN-2 compared to the Aphinity trial (56.3% vs. 24.9%, respectively).

GeparSepto randomized patients between 12 weekly cycles of paclitaxel or nab-paclitaxel followed by 4 cycles of epirubicin/cyclophosphamide. The nab-paclitaxel arm resulted in a significantly higher pCR rate compared to the paclitaxel arm which translated into a significantly better iDFS (84.0% vs. 76.3%; HR 0.66, 95% CI, 0.51–0.86; *p* = 0.002)^12^. Hence, investigating nab-paclitaxel as part of (neo)adjuvant idd chemotherapy seems reasonable from a current perspective as well.

The meta-analysis of Berruti et al.^26^ demonstrated that, in neoadjuvant trials comparing dose-dense vs. standard chemotherapy, the pCR rate seemed to be a surrogate marker on a trial level for DFS and OS only for the dose-dense regimens. The meta-analysis of Cortazar et al.^16^ failed to validate pCR rate as a surrogate endpoint for improved iDFS and OS on a trial level. This is in line with the results of GAIN-2, which was not able to confirm an OS or iDFS benefit of iddEnPC within the neoadjuvant cohort (data not shown). pCR thus remains a challenging surrogate marker for improved iDFS and OS, requiring careful consideration of regimens and subtypes analyzed.

As a result, long-term follow-up analyses are essential to prevent false conclusions that are solely based on early-reported improvements in pCR (or lack thereof). For example, in the IMpassion031 study, a promising improvement in pCR rates with atezolizumab compared to placebo could not confirm an improvement in EFS (2-year EFS 85% vs. 80%), DFS (2-year DFS 87% vs. 83%), or OS rates (2-year OS 95% vs. 90%)^27^. However, in the GeparNUEVO study, despite a nonsignificant increase in pCR rate in the durvalumab arm vs. placebo (53.4% vs. 44.2%, respectively)^28^, there were significant gains in survival with durvalumab vs. placebo (e.g., 3-year iDFS 85.6% vs. 77.2%, respectively; 3-year DDFS 91.7% vs. 78.4%, respectively; and 3-year OS 95.2% vs. 83.5%, respectively)^29^.

Even within tumor subgroups, there are differences in the ability of pCR to predict survival outcomes. In the GeparOcto study, there was no significant difference in pCR between iddEPC and PM(Cb)^30^ as well as no survival differences between both arms, yet the HR+/HER2− subgroup displayed a remarkable improvement in 4-year iDFS (77.9% iddEPC versus 62.5% PM) and OS rates (94.7% iddEPC versus 80.1% PM)^31^. In our current study, despite a better pCR achieved by the iddEPC approach, survival outcomes are comparable between both arms. This highlights the importance of reporting long-term efficacy data including subgroup analyses, which are essential to identify differences in survival outcomes.

Within the population recruited in the GAIN2 trial after amendment 3, patients with more aggressive biology and lower nodal burden were more likely to receive neoadjuvant treatment, and predominantly patients with luminal HER2- subtype and higher nodal burden were more likely to receive adjuvant treatment. Thus, biological risk detected in the initial biopsy on the one hand and the anatomical risk identified during surgery on the other hand may have been likely drivers to enroll patients in the neoadjuvant or adjuvant settings, respectively. As such, patients with a borderline or no chemotherapy indication based on initial biopsy (yet with high anatomic risk confirmed during surgery) were eligible for adjuvant chemotherapy, thereby hindering direct comparisons between both settings. Moreover, differences in risk profiles between clinically assessed nodal status within the neoadjuvant group and surgically assessed nodal status further hinder direct comparisons. Ideally, the trial would have allowed a randomization in a 2 × 2 fashion in terms of chemotherapy and setting. Furthermore, it can be hypothesized that patients with luminal HER2- subtype with higher nodal burden are well treated with adjuvant idd/tdd chemotherapy as reported earlier^7^.

The GAIN-2 trial adds knowledge on the role of idd chemotherapy in early breast cancer. Therapy with iddEnPC achieves a higher pCR rate than dose-tailored dtEC-dtD therapy, interestingly across subtypes. Due to the small sample size in the neoadjuvant cohort, the difference could not translate into an improved outcome, and selection bias might have interfered. Moreover, given that neoadjuvant treatment was allowed towards the end of trial (after amendment 3) and not from the beginning, the time lag may have also contributed to the differences in results. A further limitation is the lack of a control arm offering a comparison with the current standard sequential EC/weekly paclitaxel used as a backbone in many studies including immune checkpoint inhibitors trials.

Nevertheless, achieving a pCR can still impact survival on an individual patient level. The results are especially interesting for patients with luminal breast cancer who seem to have derived a real benefit from the idd treatment and achieved a pCR rate of 20.3%. In general, the neoadjuvant treatment with its more granular endpoint can tease out differences in risk profiles, especially where effective post-neoadjuvant therapies are available in case of residual disease.

Overall, both regimens showed comparable long-term efficacy. For high-risk patients, the idd regimen might be preferred. Their major difference lies in the clinical management with specified doses in iddEnPC and individual dose adjustment depending on toxicity in dtEC-dtD.

## Methods

### Patient selection and study design

GAIN-2 (ClinicalTrials.gov Identifier: NCT01690702; registration: September 24, 2012) was a multicenter, prospective, randomized, open-label phase III trial conducted at 136 sites in Germany as an academic collaboration between the German Breast Group (GBG) and the Breast Study Group of the “Arbeitsgemeinschaft für Gynäkologische Onkologie” (AGO-B). The GAIN-2 trial compared intense dose-dense epirubicin, nab-paclitaxel, and cyclophosphamide (iddEnPC) versus leukocyte nadir-based tailored dose-dense epirubicin/ cyclophosphamide, followed by tailored dose-dense docetaxel (dtEC-dtD), after one additional week of rest. Baseline patient and tumor characteristics as well as major inclusion criteria have been reported recently^18^. The study protocol was approved by the regional ethics committee (Ethik-Kommission bei der Landesärztekammer Hessen)/local ethics committees of participating institutes, institutional review boards, and the relevant health authorities including: Ethik-Kommission der Medizinischen Fakultät der Eberhard-Karls-Universität und am Universitätsklinikum Tübingen (Ref. 180/2012AMG2), Ethikkommission der Medizinischen Fakultät Heidelberg (Ref. Abmu-174/2012), Ethik-Kommission bei der Landesärztekammer Baden-Württemberg (Ref. B-AM-2012-061), Medizinische Ethikkommission II der Fakultät für Klinische Medizin Mannheim der Ruprecht-Karls-Universität Heidelberg (Ref. N.A.), Ethik-Kommission der Universität Ulm (Ref. 108/12), Ethik-Kommission der Albert-Ludwigs-Universität Freiburg (Ref. 129/12), Ethikkommission der Bayerischen Landesärztekammer (Ref. 7/12059), Ethik-Kommission der Medizinischen Fakultät der Ludwig-Maximilians-Universität München (Ref. 7/12059), Ethik-Kommission der Landesamt für Gesundheit und Soziales Berlin, Geschäftsstelle der Ethik-Kommission des Landes Berlin (Ref. 12/0171-ZS EK), Ethik-Kommission der Landesärztekammer Brandenburg (Ref. AS 48/2012), Ethikkommission des Landes Bremen (Ref. N. A.), Ethik-Kommission der Ärztekammer Hamburg (Ref. 118-12), Ethik-Kommission des Fachbereichs Medizin der Johann Wolfgang Goethe-Universität Frankfurt (Ref. LV 06/12), Ethik-Kommission an der Medizinischen Fakultät der Universität Greifswald (Ref. 077/2012), Ethikkommission bei der Landesärztekammer Niedersachsen (Ref. MC-098/12), Ethikkommission der Medizinischen Fakultät der Westfälischen Wilhelms-Universität Münster und der Ärztekammer Westfalen-Lippe (Ref. 2012-190-b-A), Ethikkommission der Ärztekammer Nordrhein (Ref. 2012129), Ethik-Kommission der Universität Witten/Herdecke (Ref. 35/2012), Ethik-Kommission an der Medizinischen Fakultät der Rheinischen Friedrich-Wilhelms-Universität Bonn (Ref. 083/12), Ethikkommission der Med. Fakultät der HHU Düsseldorf (Ref. MC-694), Ethik-Kommission bei der Landesärztekammer Rheinland-Pfalz (Ref. 837.150.12 (8251)), Ethik-Kommission bei der Ärztekammer des Saarlandes (Ref. 73/12), Ethikkommission bei der Sächsischen Landesärztekammer (Ref. EK-AMG-MCB-45/12-1), Ethik-Kommission der Medizinischen Fakultät der TU Carl Gustav Carus (Ref. EK 109042012), Ethik-Kommission des Landes Sachsen-Anhalt (Ref. 12/052), Ethik-Kommission der Otto-von-Guericke-Universität an der Medizinischen Fakultät (Ref. 57/12), Geschäftsstelle der Ethik-Kommission der Martin-Luther-Universität; Halle-Wittenberg (Ref. 2012-36), Ethik-Kommissionen bei der Ärztekammer Schleswig-Holstein (Ref. 041/12 (m)), Ethik-Kommission Universität zu Lübeck Med. Fak. der Universitätsklinikums Schleswig-Holstein (UKSH) Lübeck, Campus Lübeck (Ref. 12-055), Ethikkommission der Landesärztekammer Thüringen (Ref. 43182/2012/43). This study conformed to the ethical principles for clinical research involving human participants outlined in the Declaration of Helsinki. All participants provided written informed consent at enrollment.

The study was initially planned exclusively as an adjuvant trial but was opened for inclusion of patients in the neoadjuvant setting with amendment 3 (dated 28th April 2016). Until activation of amendment 3 (30th July 2016), 1973 patients had been recruited to this date, and following this amendment, 884 more patients were recruited, of whom 593 received treatment as neoadjuvant therapy. Patients were not randomized between these settings, but the decision was left to the discretion of the investigator.

Patients with centrally confirmed estrogen and progesterone receptors (ER and PgR, respectively; positivity defined as ≥1% stained cells), HER2 and Ki-67 status determined on surgically removed tissue (adjuvant patients) or from core biopsy (neoadjuvant patients) were eligible. GAIN-2 recruited only patients with high risk disease, which was defined as luminal A-like (ER and/or PgR positive, HER2-, and Ki-67 ≤ 20%) with c/pN ≥2, luminal B-like (ER and/or PgR positive, HER2-, and Ki-67 > 20%) with any involved lymph nodes, or HER2+ or triple-negative breast cancer (TNBC) irrespective of nodal status.

Patients were randomized centrally in a 1:1 ratio. The randomization was stratified based on breast cancer subtype (Luminal B-like vs HER2+/hormone receptor positive (HR+) vs Luminal A-like high-risk vs TNBC vs HER2+/HR−), and nodal status (c/pN0/1 vs c/pN2 vs c/pN3).

### Treatment

Patients in the iddEnPC arm received intense dose-dense epirubicin 150 mg/m² every 2 weeks (q2w) for 3 cycles followed by nab-Paclitaxel 330 mg/m² q2w for 3 cycles followed by cyclophosphamide 2000 mg/m² q2w for 3 cycles. In the dtEC-dtD arm, 4 courses of tailored dose-dense epirubicin (38–120 mg/m², with starting dose of 90 mg/m²) and cyclophosphamide (450–1200 mg/m², with starting dose of 600 mg/m²) were given q2w followed by one additional week of rest. Thereafter, 4 courses of tailored dose-dense docetaxel (60–100 mg/m², with starting dose of 75 mg/m²) were administered every two weeks.

Patients with HER2+ disease received trastuzumab 6 mg/kg (loading dose 8 mg/kg) every 3 weeks (q3w) simultaneously to all nP and C cycles in the iddEnPC arm and to all dtD cycles in the dtEC-dtD arm, and thereafter until completion of one year. Following amendment 3, dual blockade with additional pertuzumab 420 mg (loading dose 840 mg) q3w could be administered for patients with HER2+ tumors and only during the neoadjuvant phase (Supplementary Fig. 8). A sub-study evaluated patient preference and pharmacokinetics of subcutaneous trastuzumab in the abdominal wall versus the thigh^32^.

### Study assessments

The primary efficacy endpoint was iDFS, in addition to safety, tolerability, and quality of life. Results have already been published^18^. Secondary endpoints which we report here include the short-term endpoint pCR (ypT0/is ypN0) in the neoadjuvant treated cohort with regards to stratified breast cancer subtypes, in addition to the long-term efficacy endpoints of iDFS and OS in the neoadjuvant treated cohort as an exploratory analysis, as well as breast conservation rate. OS for the entire cohort will be reported too.

### Statistical analysis

All patients who started therapy after randomization were included in the modified intent-to-treat (mITT) population, on which the analyses are based. Median follow-up time was estimated with the inverse Kaplan–Meier method and completeness of follow-up was assessed as described by Clark et al.^33^.

OS was estimated using the Kaplan–Meier product limit method, and treatment groups were compared using the log-rank test. Cox proportional hazard models were used to estimate hazard ratio (HR) with 95% confidence intervals (CI) and to adjust for stratification factors. A Breslow–Day interaction test was performed to assess interaction between treatment arm and subgroups (biological subtype, Ki-67, nodal status, treatment setting, stromal tumor-infiltrating lymphocytes (sTILs), pCR (ypT0/is ypN0)). Analyses including the pCR rate (ypT0/is ypN0) as a covariate were performed by using a landmark analysis to avoid guarantee-time bias^34^. Only neoadjuvant patients at risk at the landmark time (4.1 months for patients in the iddEnPC arm and 3.9 months for patients in the dtEC-dtD arm) were considered in these analyses.

pCR (ypT0/is ypN0) and breast conservation were summarized as number and percent of patients for each treatment group in the neoadjuvant cohort. Two-sided 95% CI were calculated for the pCR according to Pearson and Clopper^35^, and odds ratios (OR) between treatment groups from uni- and multivariate logistic regression (adjusting for stratification factors) were reported for pCR, as well as the difference in the rates and corresponding 95% CI. No adjustment for multiple comparisons was done. The significance level was set to α = 0.05.

Data was analyzed using SAS® (Statistical Analysis Software) version 9.4 with SAS Enterprise Guide Version 7.1 and 8.3 on Microsoft Windows 10 Enterprise.

### Supplementary information

GAIN2 Supplementary information_clean

## Data Availability

Will individual participant data be available (including data dictionaries)?: Yes. What data in particular will be shared?: Individual participant data that underlie the results reported in this article, after final analysis and publication of all secondary efficacy endpoints. What other documents will be available?: Study protocol; statistical report (if necessary for the project). When will data be available (start and end dates)?: Beginning after final analysis and publication of all secondary efficacy endpoints; no end date. With whom will data be shared?: Researchers who provide translational research proposals. Proposals should be approved by the GBG scientific board. For what types of analyses?: To achieve aims in the approved proposal. By what mechanism will data be made available?: Proposal forms should be requested from trafo@gbg.de; once the application has been approved and a data transfer agreement has been signed, researchers will be given access to the data All relevant data are within the paper and its Supporting Information files. The data underlying the results presented in the study are available from GBG. Some restrictions apply due to confidentiality of patient data and materials. Since these data and materials are derived from a prospective clinical trial with ongoing follow-up collection, there are legal and ethical restrictions to sharing sensitive patient-related data publicly. Interested groups may request the “Cooperation Proposal Form” from trafo@gbg.de. Data can be requested in context of a translational research project by sending the form back to trafo@gbg.de. Translational research proposals are approved by the GBG scientific boards.
