# Supplementary material for: GAIN2 trial overall survival with intense versus tailored dose dense chemotherapy in early breast cancer
Source: NPJ Breast Cancer. 2024 Jul 30;10:66. doi: 10.1038/s41523-024-00675-x (PMC11289103; doi:10.1038/s41523-024-00675-x)
Supplement: Supplementary file 1 — GAIN2 Supplementary information_clean [file 41523_2024_675_MOESM1_ESM.pdf]

Supplementary information

Supplementary Figure 1: Consort diagram (whole study population)

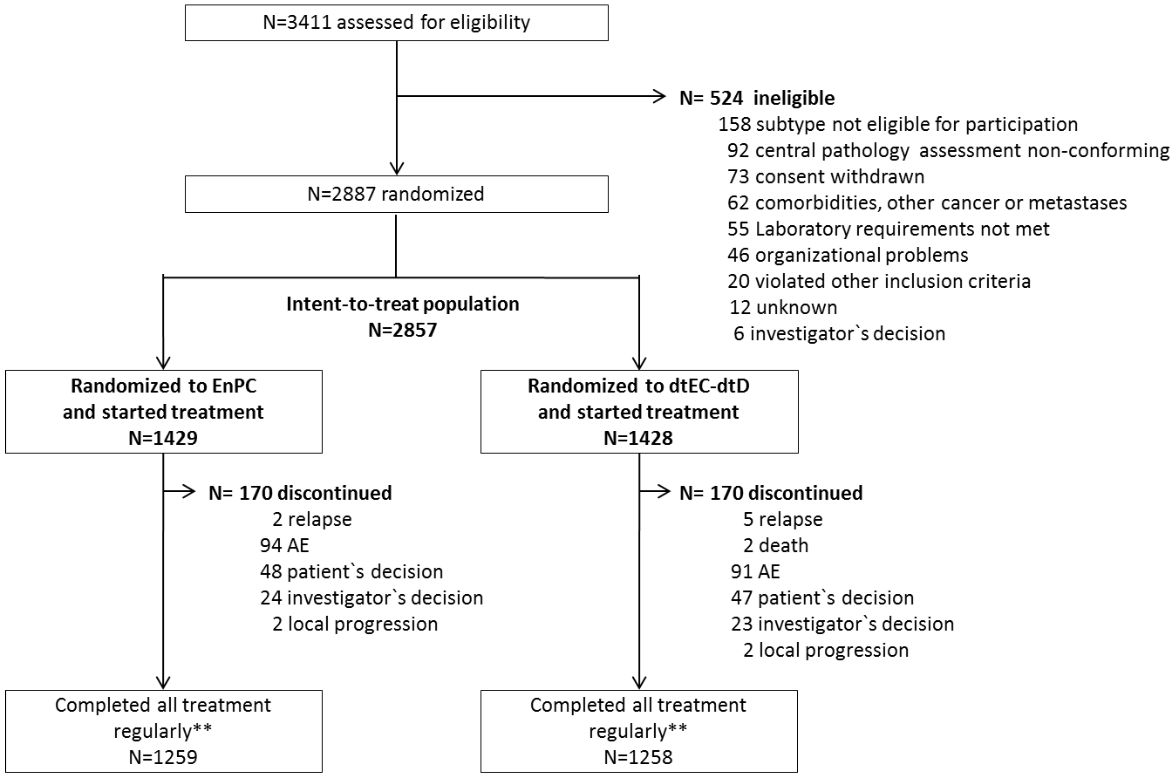

Supplementary Figure 2: Subgroup analysis of OS, entire cohort (univariate Cox regression model)

Subgroup	N patients	Hazard Ratio (95%CI)	p-Value	Test for Interaction
**Overall**	2857	1.12 (.897, 1.39)	0.321	
**Biological subtype**				0.183
Luminal A high risk	516	1.12 (.657, 1.91)	0.678	
Luminal B/HER2-	901	1.56 (1.07, 2.29)	0.021	
Triple negative	662	.934 (.639, 1.36)	0.723	
HER2+ ER+ and/or PgR+	536	.571 (.250, 1.31)	0.185	
HER2+ non-luminal	242	1.01 (.419, 2.42)	0.989	
**Ki-67**				0.432
<=20%	697	.942 (.583, 1.52)	0.806	
>20%	2160	1.17 (.913, 1.50)	0.213	
**Nodal status**				0.234
c/pN0-1	1508	1.04 (.740, 1.47)	0.809	
c/pN2	891	.938 (.622, 1.41)	0.760	
c/pN3	457	1.53 (1.02, 2.32)	0.042	
**setting**				0.615
neo-adjuvant	593	.973 (.576, 1.64)	0.917	
adjuvant	2264	1.15 (.899, 1.46)	0.270	
**sTILs**				0.739
low (0-10%)	1653	1.17 (.878, 1.55)	0.286	
intermediate (11-59%)	927	1.07 (.723, 1.59)	0.733	
high (60-100%)	269	.857 (.384, 1.91)	0.706	
**pCR (ypT0/is ypN0)**				0.648
no	306	1.15 (.624, 2.12)	0.651	
yes	278	.877 (.307, 2.50)	0.806	

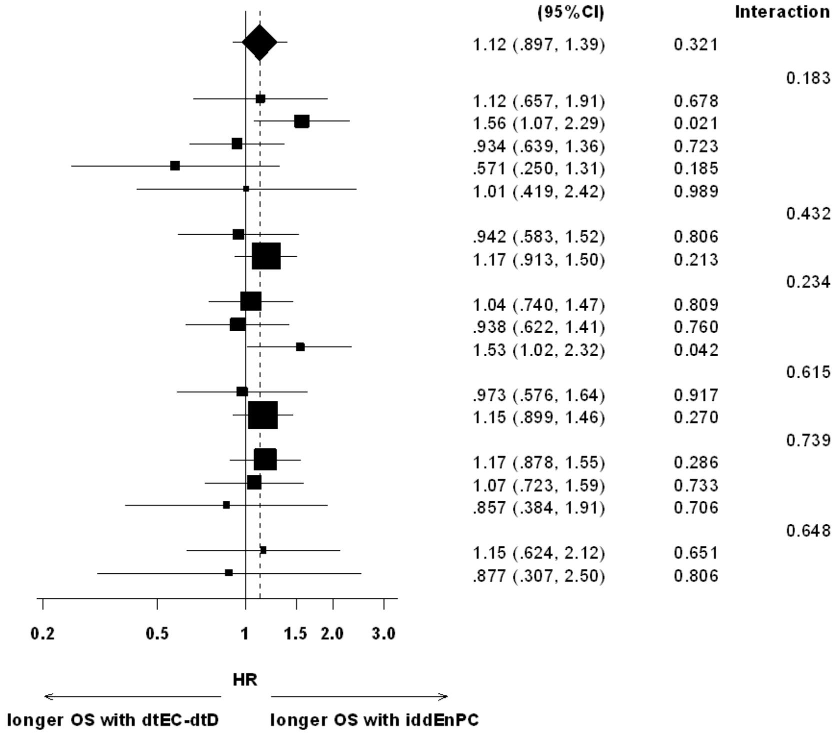

Supplementary Figure 3: Comparison of short-term treatment efficacy (ypT0/is ypN0) according to stratified and prospectively defined subgroups

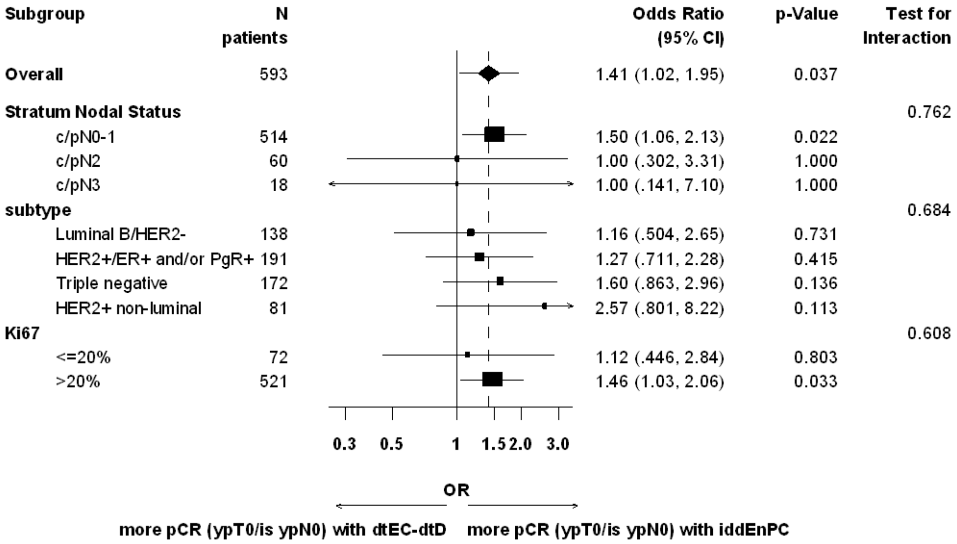

Supplemental Figure 4: Kaplan-Meier curves for iDFS rates among patients treated in the **(A)** neoadjuvant and **(B)** adjuvant settings, stratified according to tumor subtypes

(A)

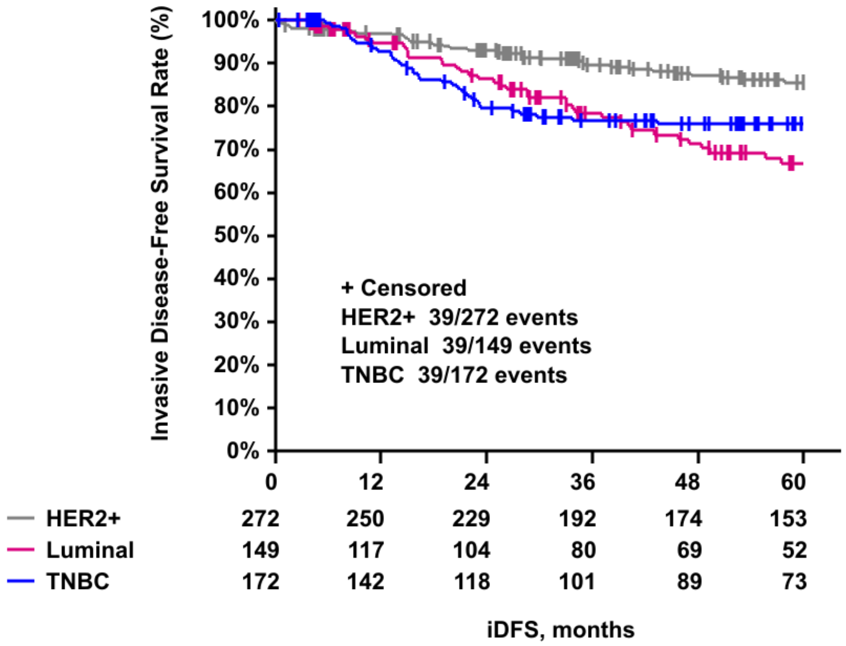

(B)

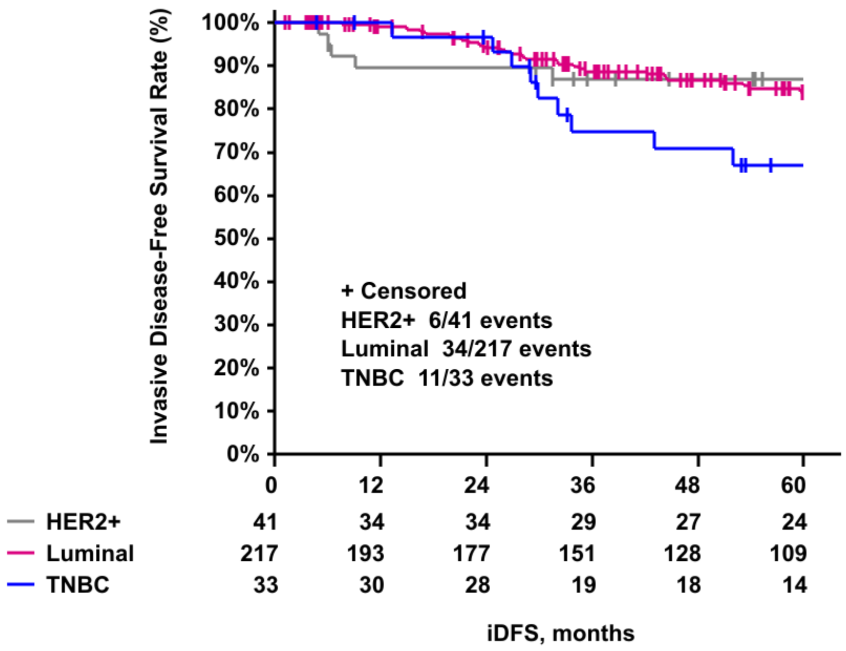

Supplemental Figure 5: Kaplan-Meier curves for OS rates among patients treated in the **(A)** neoadjuvant and **(B)** adjuvant settings, stratified according to tumor subtypes

(A)

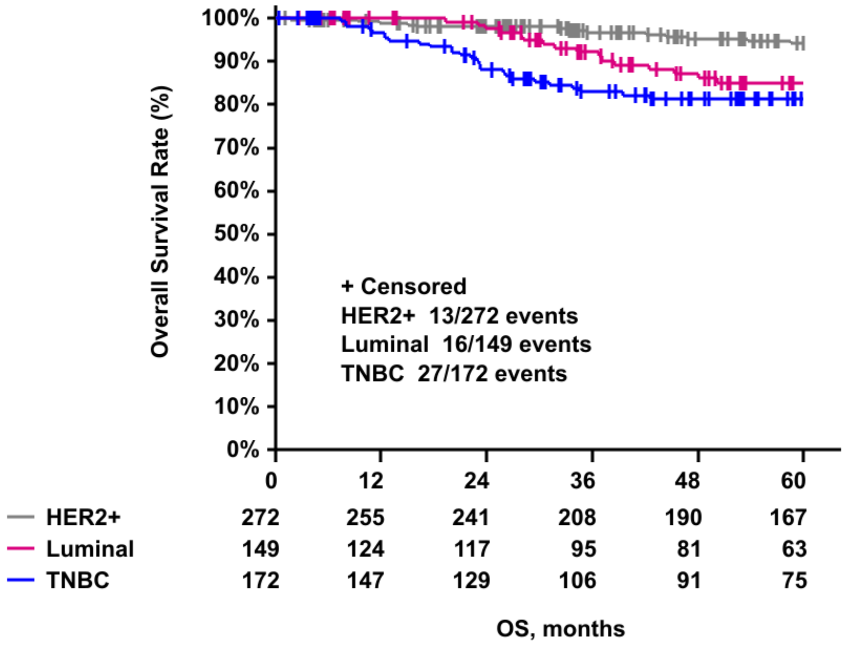

(B)

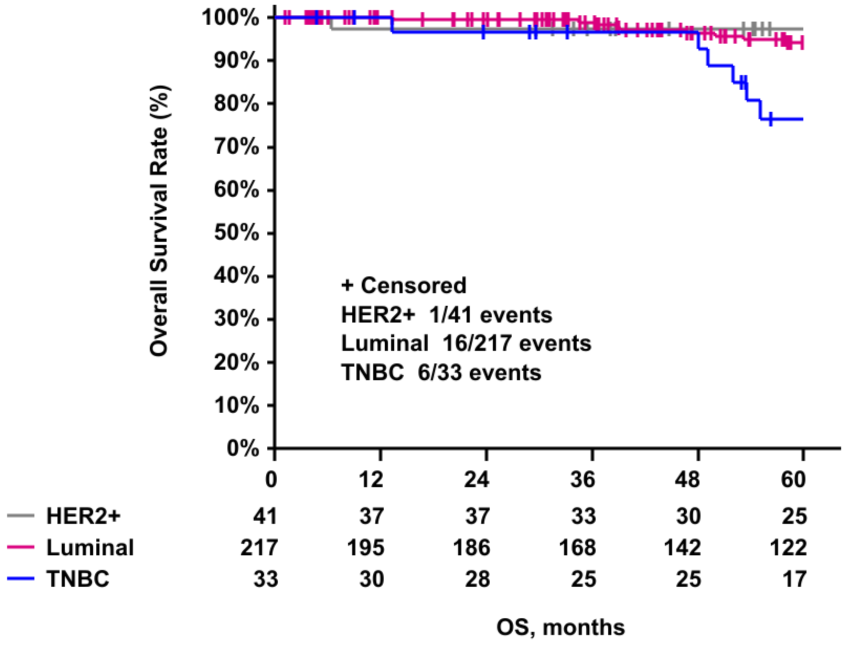

Supplementary Figure 6: Forest plot of multivariate Cox regression for OS (cohort of patients included after the third amendment)

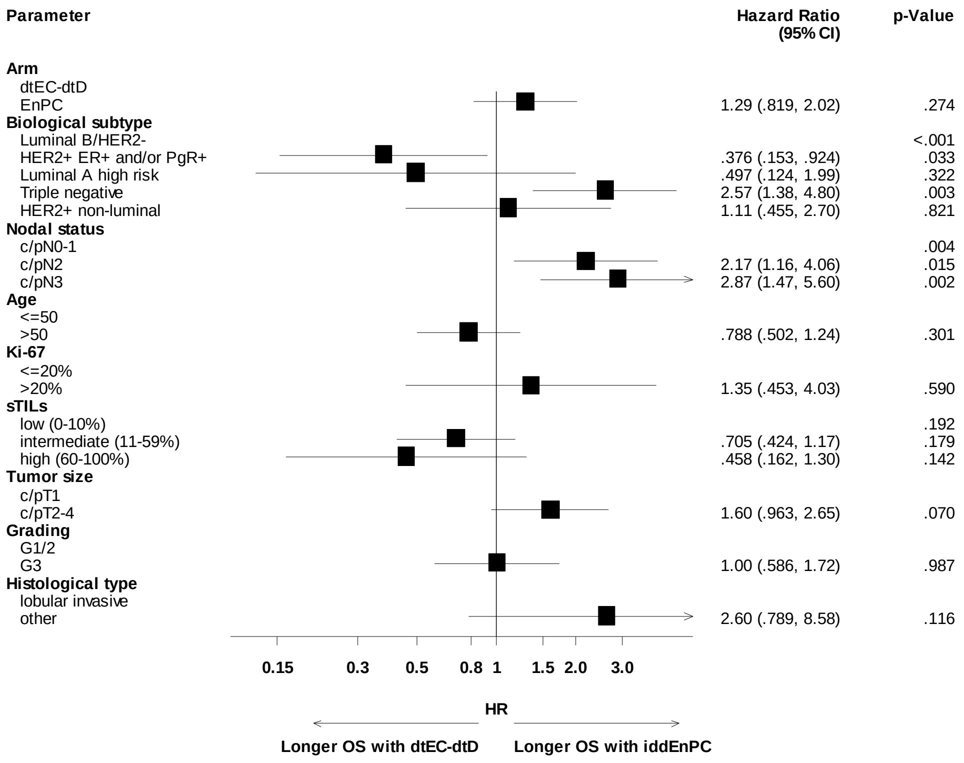

Supplementary Figure 7: Forest plot of multivariate Cox regression for iDFS (cohort of patients included after the third amendment)

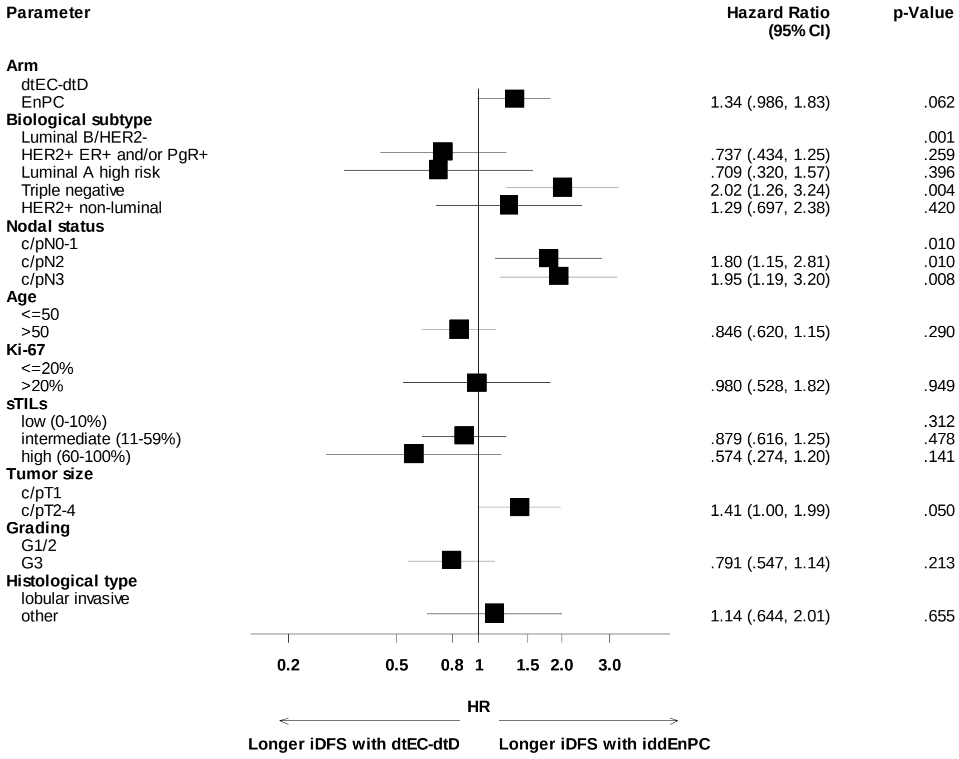

Supplementary Figure 8: Study Design

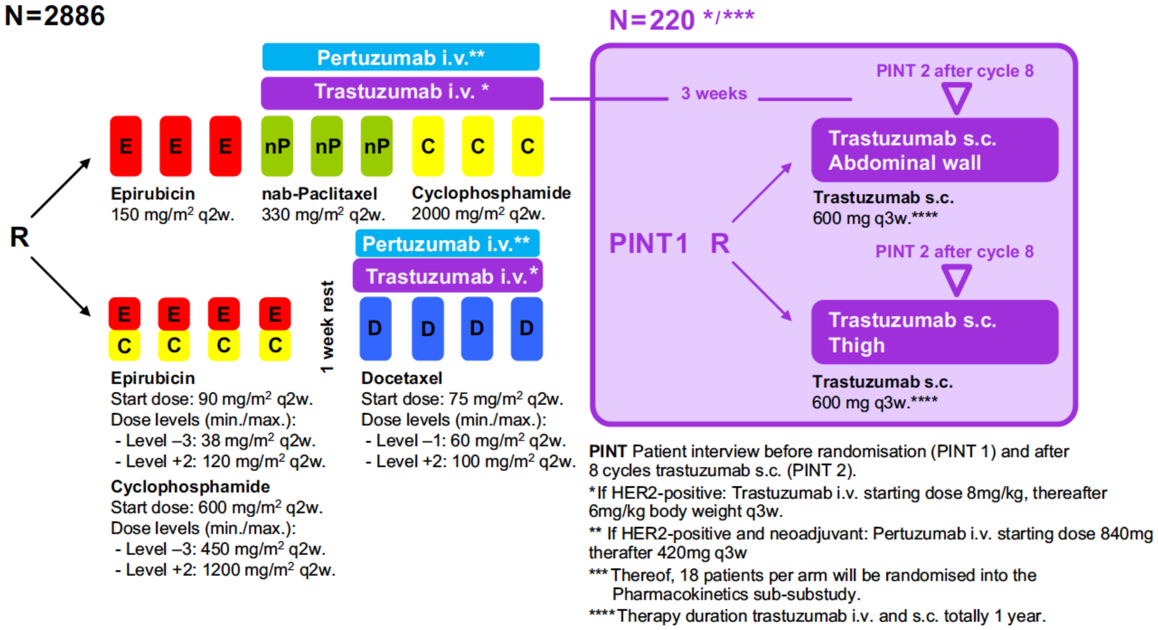

Note: It was initially planned to enroll 2,886 patients; at the end, 2,887 patients were randomized.

Supplementary Table 1: Baseline characteristics (neoadjuvant vs. adjuvant settings, all patients)

Parameter		Neoadjuvant N=593 N (%)	Adjuvant N=2264 N (%)	Overall N=2857 N (%)	*p*-value
Menopausal status	premenopausal	338 (57.0)	1140 (50.4)	1478 (51.7)	0.004
	postmenopausal	255 (43.0)	1124 (49.6)	1379 (48.3)	
Karnofsky index	Karnofsky 100%	536 (90.4)	1861 (82.2)	2397 (83.9)	<0.001
Tumor stage (all)	c/pT1	224 (37.8)	826 (36.5)	1,050 (36.8)	<0.001
	c/pT2	312 (52.7)	1,105 (48.8)	1,417 (49.6)	
	c/pT3	25 (4.2)	291 (12.9)	316 (11.1)	
	c/pT4	31 (5.2)	42 (1.9)	73 (2.6)	
Nodal status (all)	c/pN0-1	514 (86.8)	994 (43.9)	1,508 (52.8)	<0.001
	c/pN2	60 (10.1)	831 (36.7)	891 (31.2)	
	c/pN3	18 (3.0)	439 (19.4)	457 (16.0)	
ER/PgR*	ER/PR +	340 (57.3)	1,613 (71.2)	1,953 (68.4)	<0.001
	ER/PgR-	253 (42.7)	651 (28.8)	904 (31.6)	
Subtype	Luminal A high-risk	11 (1.9)	505 (22.3)	516 (18.1)	<0,001
	Luminal B	138 (23.3)	763 (33.7)	901 (31.5)	
	TNBC	172 (29.0)	490 (21.6)	662 (23.2)	
	HER2+ and/or ER/PgR+	191 (32.2)	345 (15.2)	536 (18.8)	
	HER2+/HR-	81 (13.7)	161 (7.1)	242 (8.5)	

HER2 central	positive	272 (45.9)	506 (22.3)	778 (27.2)	<0,001
	negative	321 (54.1)	1,758 (77.7)	2,079 (72.8)	
Tumor grading	G1	6 (1.0)	49 (2.2)	55 (1.9)	<0.001
	G2	215 (36.3)	1,027 (45.4)	1,242 (43.5)	
	G3	372 (62.7)	1,188 (52.5)	1,560 (54.6)	
Ki67 central	<=20%	72 (12.1)	625 (27.6)	697 (24.4)	<0.001
	>20%	521 (87.9)	1,639 (72.4)	2,160 (75.6)	
IHC4+C-score, quartiles	IHC4+C-score Q1	100 (29.6)	10 (9.6)	110 (24.9)	<0.001
	IHC4+C-score Q2	92 (27.2)	19 (18.3)	111 (25.1)	
	IHC4+C-score Q3	80 (23.7)	31 (29.8)	111 (25.1)	
	IHC4+C-score Q4	66 (19.5)	44 (42.3)	110 (24.9)	

**Central pathology, preferably based on surgical tissue (for adjuvant patients) or from core biopsy (for neoadjuvant patients), and if not available, then from local pathology.*

Supplementary Table 2: Multivariable logistic regression analysis for pCR (ypT0/is ypN0) adjusted for stratification factors

Parameter	Category	OR	95% CI	*p*-value
**Arm**	dtEC-dtD			
	iddEnPC	1.48	(1.03, 2.12)	0.033
**Subtype**	Luminal B/HER2-			<0.001##
	HER2+/ER+ and/or PgR+	5.82	(3.47, 9.75)	<0.001
	Luminal A high risk#	n.a.	n.a.	n.a.
	Triple negative	2.43	(1.44, 4.10)	<0.001
	HER2+ non-luminal	15.5	(7.73, 31.0)	<0.001
**Nodal status**	cN0-1			0.228##
	cN2	0.58	(0.28, 1.19)	0.135
	cN3	1.50	(0.53, 4.20)	0.443

CI = confidence interval; iddEnPC = intense dose-dense Epirubicin, nab-Paclitaxel, Cyclophosphamide; dtEC-dtD = dose-dense, dose-tailored Epirubicin/Cyclophosphamide - dose-dense, dose-tailored Docetaxel; mITT = modified intent-to-treat; pCR = pathological complete response; OR = odds ratio; ER = estrogen receptor; PgR = progesterone receptor.
Global p-value given in row “Subtype Luminal B/HER2-" and “Nodal status cN0-1”
procedure not attainable as all patients (N=11) fall into same category (no pCR)
global p-value

Supplementary Note

An IHC4+C like score was determined in HR-positive patients according to Cuzick et al [1] as the sum of the immunohistochemical (IHC) score and a clinical score.
According to [1], the IHC score was computed by the following formula:

$$IHC4=94.7\ [-0.1\ ER_{10} - 0.079\ PgR_{10} + 0.586\ HER2 + 0.24\ \ln(1+10\ Ki67)]$$

where IHC4 includes the four IHC markers

- ER_{10} (H score, defined as the percentage of cells staining weakly + two times the percentage of cells staining moderately + three times the percentage of cells staining strongly divided by 30 to obtain a variable with range 0-10); here, ER_{10} was replaced by the percentage of cells staining positive/10 (positive, if ER>10%)
- PgR_{10} (percentage of cells staining positive/10 (positive, if PgR>10%)
- HER2 (3+: positive; 0,1+,2+: negative; 2+ equivocal: FISH->positive if the ratio was >2)
- Ki67 (percentage of positive staining malignant cells)

Further, the clinical score given in [1] was adjusted by

$$\text{Clinical score}=100\ [0.417\ N_{1\text{-}3} + 1.566\ N_4 + 0.93\ (0.497\ T_{1\text{-}2} + 0.882\ T_{2\text{-}5} + 1.838\ T_{>5} + 0.559\ Gr_2 + 0.97\ Gr_3 + 0.13\ Age)]$$

with the variables

- nodal status ($N_{1\text{-}3}$, N_4),
- tumor stage ($T_{1\text{-}2}$, $T_{2\text{-}5}$, $T_{>5}$) (instead of using the classification of the tumor stage as in [1]:$T_{1\text{-}2}$, $T_{2\text{-}3}$, $T_{>3}$),
- local grading (Gr_2, Gr_3) and
- age.

The factor 'anastrozole' as given in the formula in [1] had to be omitted, because the variable was not available in GAIN-2 patients.
The IHC4+C- score was analyzed in risk groups, categorized in quartiles (Q) as follows:

- Q1 < 25%
- Q2 ≥ 25% and < 50%
- Q3 ≥ 50% and < 75%
- Q4 ≥ 75%

Supplementary References

[1] J. Cuzick, M. Dowsett, S. Pineda, et al. Prognostic value of a combined estrogen receptor, progesterone receptor, Ki-67, and human epidermal growth factor 2 immunohistochemical score and comparison with the genomic health recurrence score in early breat cancer. J Clin Oncol 29 (32): 4273-79, 10 Nov 2011.

CONSORT 2010 checklist of information to include when reporting a randomised trial*

Section/Topic	Item No	Checklist item	Reported on page No
		**Title and abstract**	
	1a	Identification as a randomised trial in the title	-
	1b	Structured summary of trial design, methods, results, and conclusions (for specific guidance see CONSORT for abstracts)	5
		**Introduction**	
Background and objectives	2a	Scientific background and explanation of rationale	6-7
	2b	Specific objectives or hypotheses	7
		**Methods**	
Trial design	3a	Description of trial design (such as parallel, factorial) including allocation ratio	18
	3b	Important changes to methods after trial commencement (such as eligibility criteria), with reasons	18
Participants	4a	Eligibility criteria for participants	18-19
	4b	Settings and locations where the data were collected	18
Interventions	5	The interventions for each group with sufficient details to allow replication, including how and when they were actually administered	19
Outcomes	6a	Completely defined pre-specified primary and secondary outcome measures, including how and when they were assessed	19-20
	6b	Any changes to trial outcomes after the trial commenced, with reasons	18
Sample size	7a	How sample size was determined	Ref. 18
	7b	When applicable, explanation of any interim analyses and stopping guidelines	N.A.
Randomisation:			
Sequence generation	8a	Method used to generate the random allocation sequence	Ref. 18
	8b	Type of randomisation; details of any restriction (such as blocking and block size)	Ref. 18
Allocation concealment mechanism	9	Mechanism used to implement the random allocation sequence (such as sequentially numbered containers), describing any steps taken to conceal the sequence until interventions were assigned	Ref. 18
Implementation	10	Who generated the random allocation sequence, who enrolled participants, and who assigned participants to interventions	Ref. 18
Blinding	11a	If done, who was blinded after assignment to interventions (for example, participants, care providers, those assessing outcomes) and how	N.A.
	11b	If relevant, description of the similarity of interventions	10
Statistical methods	12a	Statistical methods used to compare groups for primary and secondary outcomes	20, Ref. 18

	12b	Methods for additional analyses, such as subgroup analyses and adjusted analyses	20
		**Results**	
Participant flow (a diagram is strongly recommended)	13a	For each group, the numbers of participants who were randomly assigned, received intended treatment, and were analysed for the primary outcome	8, Fig. 1
	13b	For each group, losses and exclusions after randomisation, together with reasons	Fig. 1
Recruitment	14a	Dates defining the periods of recruitment and follow-up	Ref. 18
	14b	Why the trial ended or was stopped	N.A.
Baseline data	15	A table showing baseline demographic and clinical characteristics for each group	Table 1
Numbers analysed	16	For each group, number of participants (denominator) included in each analysis and whether the analysis was by original assigned groups	8, Fig. 1
Outcomes and estimation	17a	For each primary and secondary outcome, results for each group, and the estimated effect size and its precision (such as 95% confidence interval)	8-11
	17b	For binary outcomes, presentation of both absolute and relative effect sizes is recommended	9
Ancillary analyses	18	Results of any other analyses performed, including subgroup analyses and adjusted analyses, distinguishing pre-specified from exploratory	10-11, Supplement
Harms	19	All important harms or unintended effects in each group (for specific guidance see CONSORT for harms)	Ref. 18
		**Discussion**	
Limitations	20	Trial limitations, addressing sources of potential bias, imprecision, and, if relevant, multiplicity of analyses	16
Generalisability	21	Generalisability (external validity, applicability) of the trial findings	16
Interpretation	22	Interpretation consistent with results, balancing benefits and harms, and considering other relevant evidence	12-17
**Other information**			
Registration	23	Registration number and name of trial registry	18
Protocol	24	Where the full trial protocol can be accessed, if available	22
Funding	25	Sources of funding and other support (such as supply of drugs), role of funders	23

Citation: Schulz KF, Altman DG, Moher D, for the CONSORT Group. CONSORT 2010 Statement: updated guidelines for reporting parallel group randomised trials. BMC Medicine. 2010;8:18.
© 2010 Schulz et al. This is an Open Access article distributed under the terms of the Creative Commons Attribution License (http://creativecommons.org/licenses/by/2.0), which permits unrestricted use, distribution, and reproduction in any medium, provided the original work is properly cited.

*We strongly recommend reading this statement in conjunction with the CONSORT 2010 Explanation and Elaboration for important clarifications on all the items. If relevant, we also recommend reading CONSORT extensions for cluster randomised trials, non-inferiority and equivalence trials, non-pharmacological treatments, herbal interventions, and pragmatic trials. Additional extensions are forthcoming: for those and for up-to-date references relevant to this checklist, see www.consort-statement.org.
